# Les Crises Abdominales Des Goutteux Et Des Oxalemiques

**Published:** 1912-08

**Authors:** M. Lœper

**Affiliations:** Paris


### ABSTRACTS

### Les Crises Abdominales des Goutteux et des Oxalemiques

By PROF. M. LŒPER, Paris

*Progres Medicale*, October 28th, 1911

The gastro-intestinal symptoms of gouty individuals have been very much discussed. Prof. Lœper divides them into two classes: acute and sub-acute attacks, affecting either the stomach or the intestine, or both together at the same time. Acute attacks may consist of: gouty gastritis, with painful distension of the stomach; acute dilatation, with vomiting, cold sweat, and a tendency to syncope; spasmodic colics, painful diarrhœa, neuralgia, rather suggestive of those of lead poisoning, tabes or abdominal arterio-sclerosis. Sub-acute attacks consist of symptoms of sensitivo-motor dyspepsia, pain, alternating constipation and diarrhœa, spasmodic intestinal pains, atony of the alimentary canal, and even repeated evacuations of mucus.

These attacks seldom affect the stomach, and Prof. Lœper has only seen it once. But the intestinal form is more frequent and Prof. Lœper records a very typical case of what he terms "crise entéralgique diarrhéique," and another of "spasmodique or mucorrhéique" attack. Besides these acute forms there are minor forms resembling gastro-intestinal dyspepsia, the diagnosis of which is always confirmed by a consecutive attack of gout.

Prof. Lœper holds that these attacks are due to the action of the gouty toxins, and namely of oxalic acid, on the solar plexus: regulates gastro-intestinal secretions, and the rhythm of the movements of the alimentary canal, and its irritation causes pain, either with diarrhœa, spasms or paralysis. Therefore the abdominal attacks of gouty individuals are, or at least may be considered as attacks of toxic cœlialgia.

Nov. 4th, 1911.

Traitement du coma diabétique, by Dr. Blum, Privat-docént, Strasbourg. An effective treatment of diabetic coma must realize three essential conditions: 1° neutralise the acids and promote their elimination; 2° help to the re-mineralisation of the system and to establish the proper balance of the mineral constituents of the blood; 3° prevent the vaso-motor weakness which is common to all forms of coma.

The food must contain neither albumen nor fat; 1 or 2 pints of milk may be taken as well as 4 or 5 oz. of glucose per diem; champagne or brandy larga manu; sodium bicarbonate, 1 or 2 drachms, every 2 hours, until the urine becomes alkaline; vaso-motor and cardiac stimulants (Ringer's solution with a little adrenalin.)

Nov. 11th, 1911.

Sur le mécanisme de l'action des principaux agents diurétiques, by Prof. A. Mayor, Geneva.

An efficient diuresis must realize two conditions: it must promote the evacuation of fluids and the evacuation of poisons. Two classes of diuretics must therefore be considered, and Prof. Mayor strongly objects to the common tendency of some physicians who consider only one kind of diuretics, the "vascular diuretics."

As to the elimination of fluids, Prof. Mayor divides œdemata into two main classes: according to whether their origin is mechanical or osmotic. The first ones must be treated by digitalis, strophantus, squill, potassium salts or chloride of calcium. The second form of œdema (osmotic) must be treated by xanthic diuretics, like caffein, theobromin or theophyllin, which have the great advantage of preventing further resorption of sodium chloride; if the kidneys are too irritable, urea or lactose may be prescribed.

As to the elimination of poisons, proper diet, large doses of water, either plain water or mineral waters like Evian or Vittel, serum injections, large enemata, massage and baths are the most effective means.

Nov. 18th, 1911.

La goutte et son traitement par l'émanation du radium, by Prof. W. His, Berlin Ajter discussing the various meanings of the word "gout," Prof. His holds that this term must be restricted to a morbid entity characterised by disorders in the metabolism of nucleinic substances and by the presence in the blood of uric acid, this uric acid being independent of the patient's diet. In doubtful cases the diagnosis must be confirmed by an analysis of the patient's blood.

Inhalations of radium emanations, and namely of alpha and bêta rays, have given Prof. His excellent results which he has always confirmed by a careful examination of blood and urine. Out of 49 patients 37 have lost all their uric acid in average of 25 éances; 9 had no result and the 3 last ones had regained perfect health, although they had not lost their uric acid. The 37 successful cures seem to be definitive, uric acid having not reappeared in the blood after several months and even in one of them after a year and a half.

This extremely interesting article is full of practical points about the various apparatus required, and Prof. His' keen clinical sense will be greatly appreciated by the readers.

A. E. E. REBOUL, M. D.,
"Médecin aux eaux de Chatel-Guyon." L. R. C. P. & S., Edin., etc.,
Fellow of the Royal Society of Medicine.

